# When therapy-induced senescence meets tumors: A double-edged sword: A review

**DOI:** 10.1097/MD.0000000000042886

**Published:** 2025-06-13

**Authors:** Kang Liu, Haijin Huang, Minhong Zhang, Siming Chen, Yitao Yang, Chunyun Fang, Xiaojuan Zhong

**Affiliations:** a Breast Surgical Department, Ganzhou People^’^s Hospital, Ganzhou, Jiangxi Province, P.R. China; b The First Affiliated Hospital Of Gannan Medical University, Ganzhou, Jiangxi Province, P.R. China.

**Keywords:** cancer, cellular senescence, senescence-associated secretory phenotype, therapy-induced senescence, tumor microenvironment

## Abstract

The tumor microenvironment (TME) significantly influences tumor development, progression, and clinical outcomes. Therapy-induced cellular senescence is a fundamental process affecting the microenvironment. This review summarizes the characteristics of therapy-induced cellular senescence, its beneficial and detrimental effects on the TME, and the underlying mechanisms contributing to its dual effects. It further elaborates on optimizing the beneficial aspects of therapy-induced cellular senescence while concomitantly mitigating its adverse effects in the treatment of tumors and prevention of recurrence. Finally, potential interventions, including antiaging drug therapies, senescence inducers, senescence clearance agents, and inhibition of adverse senescence-associated secretory phenotype (SASP) production were explored to inhibit the harmful SASP induced by therapy, with the aim of limiting the production of detrimental SASP in the TME, thereby reducing the risk of tumor recurrence.

## 1. Introduction

As is well documented, tumor tissue is located in a complex internal environment containing tumor cells, noncancer cells, and the surrounding extracellular matrix. A wide range of immune cells, cancer-associated fibroblasts, endothelial cells, stromal cells, and different cell types that differ according to the tissue type, including adipocytes and neurons, constitute the tumor microenvironment (TME).^[1]^ These non-tumor cells were previously regarded as bystanders in tumorigenesis but have now been established to have crucial contributions to the development of cancer. Therefore, these are potential therapeutic targets.^[2]^

The cellular makeup and functional condition of the TME vary significantly based on the anatomical site of tumor development, inherent properties of cancer cells, tumor stage, and patient-specific factors.^[3]^ The TME undergoes fundamental alterations during every stage of cancer development, including tumor origin, progression, invasion, infiltration, metastasis, and proliferation.^[4]^ Cells within the TME can either contribute to the tumor-suppressive microenvironment or promote tumor progression. Thus, there is a pressing need to elucidate the complex interactions between endogenous tumor cells, exogenous factors, and systemic mediators.^[5]^

## 2. Cellular senescence

In 1961, American cell biologist Leonard Hayflick first introduced the concept of cellular senescence.^[6]^ They discovered that healthy human cells could not replicate indefinitely. After repeated divisions in vitro, they entered an irreversible cell cycle arrest referred to as senescence, a phenomenon also termed the Hayflick limit.^[7]^ The accumulation of senescent cells with impaired replication is a hallmark of organismal aging. Cellular senescence can also be triggered by various stressors that cause DNA damage, including oxidative stress, genotoxic radiation, and oncogene activation, known as “stress-induced senescence.” Senescent cells are characterized by increased lysosomal activity, chromatin rearrangement, metabolic dysregulation, resistance to apoptosis, DNA damage, and the senescence-associated secretory phenotype (SASP).^[8]^ Inducers of cellular senescence include DNA damage, telomere shortening, hypoxia, nutrient deprivation, cellular stress, oncogene activation, mitochondrial dysfunction, and possible stimulation through cellular metabolism, apoptosis regulation, unfolded protein response, and DNA damage response.^[9]^

Senescent cells generated by various stimuli exhibit uniform characteristics, such as consistent growth arrest, limited resistance to apoptosis, ongoing DNA damage signaling, modified heterochromatin structure, mitigated lamin B1 levels, and increased expression of cell cycle inhibitors such as p16INK4a (encoded by the INK4a/ARF locus, also referred to as Cdkn2a), p21Cip1/Waf1 (encoded by Cdkn1a), and senescence-associated β-galactosidase (SA-β-gal). Multiple components, including pro-inflammatory cytokines, chemokines, matrix metalloproteinases (MMPs), bioactive lipids, noncoding nucleotides (miRNA and mitochondrial DNA), vesicles, and growth factors, are released by senescent cells and collectively referred to as SASP.^[10]^

## 3. Therapy-induced senescence (TIS)

At present, it is widely recognized that conventional treatments for diseases such as cancer, including chemotherapy and radiation therapy, induce high levels of DNA damage in patient cells and lead to the secretion of numerous SASP factors, thereby culminating in cellular senescence. This phenomenon is referred to as “therapy-induced senescence (TIS).”^[11]^ Chemotherapy, radiation therapy, and targeted therapies can promote cellular senescence in the TME, affecting both cancer cells and their surrounding stromal cells.^[12]^ Prior investigations have shown that 31% to 66% of cancer tissues subjected to different types of chemotherapy display TIS. In addition, TIS has been quantified not only in malignant and nonmalignant fractions of tumor tissues but also in healthy tissue specimens after chemotherapy or radiation therapy.^[13]^ TIS is a common response to traditional cancer treatments. It was once considered a beneficial outcome of cancer therapy, and is currently regarded as a potential target for developing novel therapeutic approaches to inhibit cancer cells.^[14]^

In addition, TIS actively releases a range of immune modulators, inflammatory cytokines, growth factors, chemokines, and proteases, collectively known as SASP. The SASP comprises pro-inflammatory cytokines, including interleukin-6 (IL-6), interleukin-8, and interleukin-1ɑ, and additional chemokines that specifically attach to the interleukin-8 receptor C-X-C pattern chemokine receptor 2 (CXCR2) along with CXCL-2/-3/-5^[15]^ (Table 1).

**Table 1:** The function of SASP.

SASP	Mechanisms promoting tumor mechanisms	Mechanisms inhibiting tumorigenesis
PGE2	Antitumor immunity of CD8 + T cells	
CCL2		(1) Myeloid cell recruitment; (2) MDSC differentiation
CCL5		Lymphocyte recruitment
IL-6	Recruiting MDSCs, influencing DC differentiation, inhibiting antitumor T cell responses	Recruiting macrophages and NKT cells
IL-8		Promoting angiogenesis, invasion of neutrophils, and MDSCs
IL-1α	Macrophage immune surveillance	Regulating the pro-tumorigenic effects of IL-6 and IL-8 and inducting tumor survival factors
TGF-β1		Inflammation, induction of ROS-mediated DNA double-strand breaks
TGF-β3	Inhibiting angiogenesis	
CCL5	Recruiting TILs to eliminate cancer cells	
TNFα		Inducing T cell senescence
IFNɣ	Inducing M1 macrophage differentiation	
IFNα		Inducing CD8 + T cell senescence
MMPs	Cancer invasion promotion	
ECM	Promoting cancer metastasis	
VEGF	Promoting cancer metastasis	
PAI-1		Supporting cell cycle arrest and preventing cancer progression
IGFBP7		Supporting cell cycle arrest and preventing cancer progression
Exosomes	Associated with tumor promotion, immune escape, drug resistance, and antiapoptotic features	

CCL2 = chemokine (C-C motif) ligand 2, CCL5 = chemokine (C-C motif) ligand 5, ECM = extracellular matrix, FGFR4 = fibroblast growth factor receptor 4, IFNγ = interferon gamma, IFNα = interferon alpha, IGFBP7 = insul-inlike growth factor-binding protein 7, MMPs = matrix metalloproteinases, PAI-1 = plasminogen activator inhibitor-1, PGE2 = prostaglandin E2, TNFα = tumor necrosis factor alpha, VEGF = vascular endothelial growth factor.

### 3.1. Chemotherapy-induced cellular senescence

Chemotherapy is a conventional method for inducing senescence in both tumor and non-tumor cells. Indeed, some chemotherapeutic agents can stimulate senescence in primary murine and human cells.^[16]^ Classic inducers of senescence in tumor cells and numerous cell line models include topoisomerase toxins, including etoposide and camptothecin, which induce senescence in immortalized and primary tumor cells. At the same time, alkylating agents such as the anthracycline antibiotic doxorubicin are used to treat various cancers.^[17,18]^ For example, doxorubicin penetrates DNA and inhibits topoisomerase II by repairing the DNA double-strand breaks produced by the enzyme to relax the torsional strain, thus inducing senescence in fibroblasts. Additionally, drugs, such as melphalan and cyclophosphamide, induce senescence in the blood cells of mice. Temozolomide, a well-established chemotherapeutic drug for the treatment of brain cancer, can also induce a senescent phenotype in glioblastoma cell lines.^[19]^ Cisplatin induces cellular senescence in ovarian, lung, liver, and nasopharyngeal tumor cell models.^[20,21]^

Chemotherapy may result in either apoptosis or senescence, contingent upon the intensity of its effect on tumor suppressor factors such as P16 and P53, or on the duration and strength of drug exposure. More precisely, higher dosages of chemotherapy generally cause cell death, whereas intermediate levels may induce senescence.^[22]^

### 3.2. Radiation-induced cellular senescence

Radiotherapy is a popular treatment modality for cancer and is a significant inducer of cancer cell senescence. Preclinical studies have shown that ionizing radiation (IR) induces changes in senescence markers, including SA-β-gal, p53, p21, and various SASP factors both in vitro and in vivo.^[23,24]^ According to previous studies, eliminating radiation-induced senescence in the brain TME can lower the risk of glioblastoma recurrence.^[25]^ Radiation has also been reported to stimulate senescence in breast cancer cells^[26]^ and elevate the number of SA-β-gal-positive H1299 non-small cell lung cancer cells.^[27]^

Similar to chemotherapy, radiation-induced senescence in immune cells can suppress effective immune responses and result in the persistence of normal senescent cells, eventually leading to chronic inflammation, fibrosis, impaired tissue function, and accelerated aging. In contrast to the systemic consequences of chemotherapy-stimulated senescence, radiotherapy is administered directly to tumor locations, thereby reducing harm to noncancerous tissues.^[28]^

### 3.3. Targeted therapy-induced cellular senescence

The existing literature indicates that the use of targeted treatments that block CDK, NOTCH, CK2, MDM2, JAK2, and SKIP2 may induce growth arrest and senescence in cancers with diverse genetic backgrounds. Presently, palbociclib, an inhibitor of CDK4/6, is considered as one of the most significant senescence-inducing compounds.^[29]^ Palbociclib, a selective CDK4/6 inhibitor, stimulates senescence in gastric cancer cells.^[30]^ By combining CDK4/6 inhibitors or aurora kinase A inhibitors with MDM2 inhibitors, senescence can be triggered, followed by SASP-mediated tumor infiltration by cytotoxic T cells and immunological clearance of senescent tumor cells.^[31]^

Lapatinib therapy induces phenotypic alterations in HER2-positive cell lines that are characteristic of a senescent phenotype and demonstrate potent SA-β-gal activity. Lapatinib-stimulated senescence is linked to elevated levels of p15 and p27 but is not dependent on p16 or p21 expression.^[32]^ Generally, TIS establishes a novel mechanism of action for HER2-targeted drugs and may promote resistance development.^[33]^ INX-315, a specific inhibitor of CDK2, may stimulate cell cycle arrest and senescence in solid malignancies.^[34]^ Therefore, developing senescence-based therapies targeting tumor cells is vital for improving outcomes associated with tumor recurrence.

## 4. Impact of TIS on the TME

Tumor disease development, metastasis, medication resistance, and immunological evasion were all significantly influenced by the TME. It was used to assess the overall clinical outcomes of cancer treatment. Pharmacological induction may induce senescence in both malignant and nonmalignant tumor cells. In brief, TIS may affect the long-term prognosis of cancer by affecting TME.

**Figure 1.**
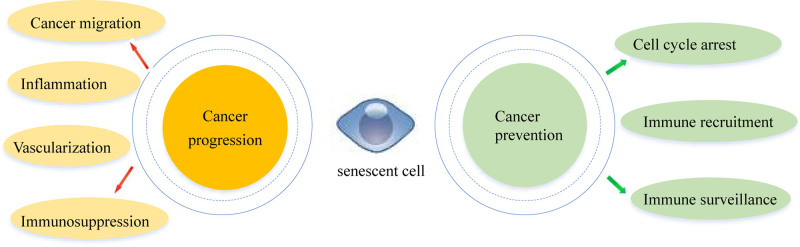
The function of senescence cells. TIS primarily induces inflammation, increases cancer migration, secretes immunosuppressive factors, and promotes angiogenesis, all of which lead to cancer progression. However, TIS also induces cell cycle arrest, promotes immune recruitment, and enhances immune surveillance, thereby contributing to cancer prevention. TIS = therapy-induced senescence.

Significantly, the process of senescence triggers the activation of many pleiotropic cytokines, chemokines, growth factors, and proteases, which are together referred to as the SASP. This activation results in continuous arrest of tumor cells and remodeling of the tumor immune microenvironment.^[35]^ On the one hand, SASP can promote antitumor immunity and therapeutic efficacy; on the other hand, it can promote the infiltration of immune-suppressive cells, contributing to immune evasion by tumor cells.^[36]^ However, the specific effects of SASP in this context remain unclear (Fig. 1).

### 4.1. Adverse effects of TIS

**Figure 2.**
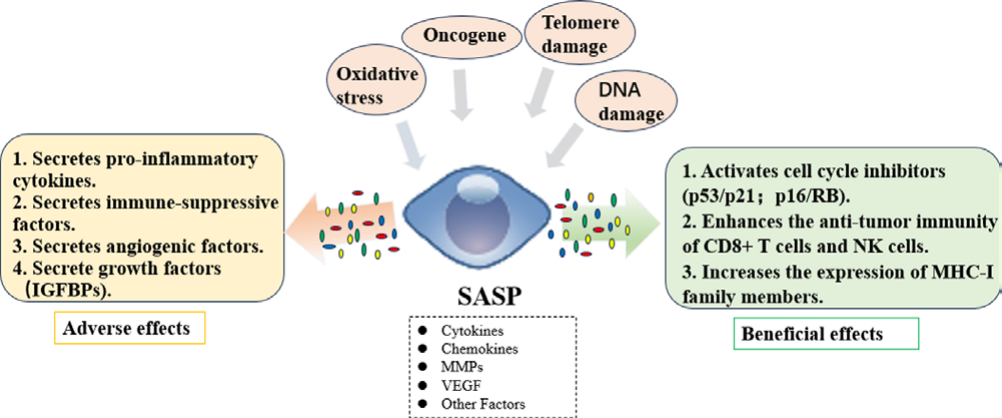
The dual effects of SASP. Adverse effects: secretes pro-inflammatory cytokines, secretes immune-suppressive factors, secretes angiogenic factors, and secretes growth factors (IGFBPs). Beneficial effects: activates cell cycle inhibitors (p53/p21; p16/RB), enhances the antitumor immunity of CD8 + T cells and NK cells, and increases the expression of MHC-I family members. MHC-I = major histocompatibility complex class I, SASP = senescence-associated secretory phenotype.

Senescence is one of the mechanisms underlying antitumor responses. However, existing evidence suggests that TIS may contribute to treatment failure by inducing tumor dormancy and triggering recurrence. Senescence allows tumor cells to evade cytotoxic effects and extend their survival in a dormant state. However, these cells may regain self-renewal capabilities, leading to disease relapse.^[37]^ Gopal et al^[38]^employed single-cell RNA sequencing and fluorescent reporter assays representing different transcriptional states to model cell state dynamics and TIS at the single-cell level, and determined that cell phenotype transitions are influenced by various senescence states during chemotherapy, determining the persistence of quiescent tumor programs. Over time, some of these cells may return to a proliferative state, thereby conferring drug resistance and leading to an invasive relapse (Fig. 2).

Post-chemotherapy, SASP predominantly drives inflammation by secreting pro-inflammatory cytokines, suppressing immune cell infiltration, evading immune surveillance by releasing immunosuppressive factors, and promoting tumor growth by secreting growth factors and enhancing angiogenesis. This leads to increased invasion and metastasis of adjacent nonsenescent tumor cells.

#### 4.1.1. SASP secreted by senescent cells promotes tumor proliferation and metastasis in various cancers

SASP factors synthesized by senescent cells can promote tumor proliferation and metastasis. SASP-induced inflammation may adversely affect cells and trigger cancer cell proliferation. For example, the SASP factors IL-1α, IL-6, TGF-β, CXCL-1, and CXCL-2, released by oncogene-stimulated human fibroblasts, may either promote senescence in an autocrine manner or trigger senescence in nearby cells via paracrine activities. IL-1α affects cells through autocrine signaling, induces inflammation, and enhances the secretion of IL-6/-8.^[39]^

Research has revealed that tiny extracellular vesicles (extracellular vesicle SASP) generated by oncogene-stimulated fibroblasts and MCF7 cancer cells treated with palbociclib trigger identical paracrine signaling.^[40]^ Moreover, regarding SASP during chemotherapy, MMPs cleave NKG2D ligands on the surface of senescent tumor cells, thereby aiding in NK cell surveillance evasion and allowing their persistence after cancer treatment.^[41]^ Prior investigations have shown that the administration of chemotherapy to patients with prostate cancer and xenograft models results in the release of SASP factor amphiregulin from senescent stromal cells. This factor stimulates the expression of immunological checkpoint programmed death ligand 1 in tumor cells, eventually causing suppression of T cells and resistance to chemotherapy.^[42]^

#### 4.1.2. SASP and its role in cancer progression

The SASP incorporates growth factors, including pro-angiogenic vascular endothelial growth factor, which may stimulate cancer-linked angiogenesis. Furthermore, MMP production by senescent cells may facilitate cancer cell proliferation and trigger vascular endothelial growth factor-dependent formation of blood vessels. Furthermore, SASP may stimulate epithelial-mesenchymal transition, thereby promoting cancer vascularization and tumor proliferation.^[43]^ Studies have noted that Senescent fibroblast-secreted factors in the TME can support tumor angiogenesis in xenograft models and enhance the metastatic and proliferative abilities of premalignant epithelial cells.^[44]^ Post-treatment, rare but spontaneous TIS escape can be observed in breast, non-small cell lung, colon, and ovarian cancers cells.^[39]^

#### 4.1.3. Systemic effects and side effects of chemotherapy-induced senescence

Chemotherapy acts systemically, resulting in the accumulation of senescent cells in non-tumor areas. However, its excessive accumulation can lead to severe adverse effects. Senescent cells, via SASP, secrete pro-inflammatory cytokines and chemokines, which interfere with stem cell activity and tissue regeneration, thereby altering the systemic equilibrium. The increase in the number of senescent cells with advancing age may potentially exacerbate age-linked disorders such as Alzheimer disease and the advancement of cancer. The use of anti-senescence drugs to eliminate senescent cells has the potential for positive outcomes in the treatment of these conditions.^[45]^

### 4.2. Beneficial effects of TIS

Senescent cells exhibit functional loss or halted proliferation, whereas tumor cells are characterized by excessive proliferation and increased metabolic rates. Mounting evidence suggests that senescence involves mechanisms that prevent cancer development and promote tumorigenesis (Fig. 2).

#### 4.2.1. SASP-induced cell cycle arrest

Senescence can also serve as a potent antitumor mechanism by preventing cell proliferation. Specific components of the SASP pathway, including IL-6/-8, plasminogen activator inhibitor-1, and insulin-like growth factor-binding protein 7 (IGFBP7), induce cell cycle arrest throughout senescence.^[46]^ IGFBP4 and IGFBP5 participate in senescence induction by regulating the IGF signaling pathway and engaging in cellular stress response mechanisms. Their synergistic roles in tissue growth and development as well as their interactions in stress responses may represent one of the key mechanisms underlying the decline in cellular function during aging.^[47,48]^ Cell cycle arrest is primarily mediated by the activation of one or both of the tumor suppressor pathways p53/p21^cip^ and p16^INK4a^/Rb.^[49]^ The p53-p21 and p16-Rb pathways play crucial roles in stress-induced senescence. The p53-p21 pathway is primarily responsible for initiating senescence, while the p16-Rb pathway plays a significant role in maintaining the long-term senescent state.^[6]^ They differ in cell type, tissue distribution, and physiological functions. Moreover, under various stress conditions, the p53/p21^cip^ and p16^INK4a^/Rb pathways work synergistically to induce cellular senescence. For instance, in DNA damage-induced senescence, the p53-p21 pathway is activated first, followed by activation of the p16-Rb pathway, which helps maintain long-term cell cycle arrest.^[50]^ The synergistic action of these 2 pathways is critical for determining cell fate under stress conditions, including cell cycle arrest, senescence induction, and SASP.^[51]^ Collectively, these mechanisms determine the cellular response to stress and influence tissue homeostasis and disease progression.

#### 4.2.2. SASP promotes immune recruitment

Immune cell recruitment is facilitated by the SASP, which encompasses myeloid-derived suppressor cells, regulatory T cells, M2 macrophages, and N2 neutrophils. These cells can enhance immune responses, thereby promoting tumor regression.^[18,52]^ Experimental evidence has shown that tumor IR may trigger RB-dependent senescence, thus augmenting the immunological surveillance of osteosarcoma by natural killer T cells.^[53]^ In pancreatic tumor models, the combination of IR with PARP inhibitors inhibits DNA repair, thereby exacerbating DNA damage and resulting in the production of inflammatory SASP factors including CCL5, IFN-β, and CXCL9/10/11. These factors promote macrophage proliferation and enhance antitumor immunity via CD8 + T and NK cells.^[54]^

#### 4.2.3. TIS enhances immune surveillance

The SASP can ameliorate IFNγ responses, thereby promoting antigen presentation and immune surveillance. Additionally, TIS upregulates antigen-processing and presentation-linked gene expression.^[55]^ The SASP enables damaged senescent cells to communicate their impaired state to neighboring cells or stimulate the immune system to promote their clearance. Various drugs (e.g., doxorubicin, Palbociclib, and Nutlin-3A) induce senescence in cancer cells by increasing the expression of classical major histocompatibility complex class I family members and antigen-processing-linked genes.^[56]^ It is worth emphasizing that CDK4/6 inhibitors induce marginally higher expression of antigen presentation-related genes (HLA-F/-B/-C, TAP1, and B2M) than do DNA-damaging agents. The IFN signaling pathway triggers the expression of target genes associated with antigen presentation^[57,58]^ (Fig. 1).

## 5. Antiaging-based cancer therapies

### 5.1. Senolytic therapy

#### 5.1.1. Therapeutic effects

Senolytic drugs selectively eliminate senescent cells by targeting the key pathways that sustain their survival. The first reported senolytic therapy combined dasatinib with quercetin.^[59,60]^ The former is a tyrosine kinase inhibitor, whereas the latter is a natural flavonoid that activates estrogen receptors and inhibits PI3 kinase. Studies have shown that this combination effectively eliminates senescent fibroblasts, prolongs survival rates, and delays the onset of age-related diseases in mice.^[61]^ A proven senolytic drug, the BCL-XL/BCL-2 inhibitor ABT-263 (navitoclax), induces apoptosis in senescent prostate cancer cells, thereby inhibiting proliferative recovery and tumor cell development.^[18]^ According to a previous study, radiation therapy induces senescence in non-tumor brain cells to promote glioblastoma recurrence, implying that senolytic therapy can inhibit the growth and invasiveness of recurrent glioblastoma. Recently, pioneering studies have indicated that eliminating senescent cells can prolong survival, enhance health, and relieve age-related diseases.^[62,63]^

#### 5.1.2. Potential side effects

Senolytic therapy has some side effects. (1) Toxic reactions: certain antiaging drugs, such as Navitoclax, have shown some toxicity in clinical trials, such as thrombocytopenia and neutropenia. (2) Impact on the immune system: although antiaging drugs can improve immune senescence, their long-term use may affect the balance of the immune system, such as weakening immune surveillance.^[64]^ (3) Potential tissue damage: although antiaging drugs can clear senescent cells, excessive clearance may decrease the number of cells in certain tissues, affecting the normal function of the tissue.^[65]^ Therefore, although antiaging drugs have the potential to delay aging and treat chronic diseases, their impact on healthy tissues needs to be carefully evaluated.

### 5.2. Combination therapy with senescence inducers and senolytics

The concept of a “one-two punch” approach for cancer treatment has been proposed, wherein the initial step involves the use of a drug to stimulate senescence in cancer cells and the second step involves the use of another drug (such as a senolytic) to eliminate senescent cancer cells. Cancer therapies (the “first punch”) stimulate senescence in both tumors and healthy tissues. Senescent cells are subsequently cleared through immune surveillance but may accumulate following cancer treatment.^[64]^ Therapy-stimulated senescent cells are specific and dynamic with diverse biomarkers, cellular plasticity, and SASP expression.

The utilization of senolytics (the “second punch”) to selectively clear senescent cells can prevent tumor recurrence, metastasis, and resistance.^[66]^ Similarly, selective elimination of senescent cells from healthy tissues can prevent, alleviate, and treat treatment-induced side effects, thereby restoring tissue homeostasis. Despite the combination of traditional anticancer drugs and senolytics remaining in the early stages of research, reports have validated their effectiveness in suppressing tumor cells. For example, a study performed by Lewińska et al^[67]^ revealed that inducing senescence in breast cancer cells (MDA-MB-231 line) and breast epithelial cells (HMEC) with etoposide, followed by treatment with a quercetin derivative, led to the lysis of breast cancer cells without affecting normal breast epithelial cells.^[68]^ Another study found that a selective fibroblast growth factor receptor 4 inhibitor (BLU9931) induced senescence in pancreatic ductal adenocarcinoma cells, which were then treated with quercetin, resulting in tumor cell death.^[69]^ Meanwhile, DeMaria et al developed a mouse model using genetic ablation of senescent cells to show that eliminating senescent cells after chemotherapy hinders the suppression of bone marrow, safeguards against heart malfunction and cancer recurrence, and enhances physical activity and strength.^[70]^ Thus, an additional benefit of using senolytics in cancer therapy is the possibility of reducing the adverse effects of cancer treatment.

### 5.3. SASP inhibitors and senomorphics

Most of the tumor-promoting effects of senescent cells are closely linked to SASP. Therefore, effectively targeting the detrimental components of the SASP, while potentially preserving the immune-activating functions of senescent cells, could serve as a viable adjunctive therapy. JAK signaling pathway inhibitors have been reported to alleviate inflammation and age-related frailty in aged animals.^[71]^ Notably, selective JAK1 and JAK2 inhibitors have shown promising results in enhancing treatments for bone fibrosis.^[72]^ In addition, inhibitors of the p38 pathway can suppress the SASP, thereby reducing bone loss and metastasis in TIS breast cancer mouse models. The NF-κB-mediated signaling pathway is a major regulator of the pro-inflammatory effects of SASP.^[73]^ Notably, metformin partially inhibited the movement of NF-κB components into the nucleus and subsequent activation of the promoters of target genes. This hinders the expression of several SASP factors, which may contribute to its antiaging properties.^[74]^ Preclinical studies have shown that mTOR inhibitors such as rapamycin have the potential to induce an aging environment, decrease NF-κB activity, inhibit pro-inflammatory SASP, and limit the growth-promoting effects of senescent fibroblasts in prostate tumors.^[62,75]^ However, SASP inhibitors may require long-term administration given that their effects might diminish after discontinuation, which is a consideration for patient adherence to clinical treatments.

Senomorphics modulate the signaling pathways or metabolic processes of senescent cells, altering the types and levels of secreted cytokines and chemokines. For instance, by inhibiting certain signaling pathways, they reduce the secretion of pro-tumor inflammatory factors while preserving or enhancing signals that facilitate immune cell recruitment.^[76]^ This approach mitigates the harmful effects of pro-tumor SASP factors while retaining the tumor-suppressive functions of senescent cells.^[77]^ As an emerging intervention strategy, senomorphics offers novel insights into cancer treatment. They may be particularly suitable for therapeutic scenarios in which the beneficial functions of senescent cells need to be preserved, such as in combination with immunotherapy. Future research is needed to further explore the specific mechanisms of action, targets, and potential clinical applications.

## 6. Summary

Although cellular senescence is a common outcome of cancer treatment, senescent cells can have varying effects on the TME. The current novel therapeutic agents include metformin, NAD+/Sirt activators, GLP-1 receptor agonists, and rapamycin. Advances in aging biomarkers (e.g., DNA methylation, glycation, metabolomics, and proteomics) may enable the clinical translation of antiaging interventions. Senescence is a double-edged sword of cancer research. On one hand, it plays a decisive role in antitumor defense; on the other hand, cellular senescence and SASP can confer resistance to treatment, thereby leading to disease recurrence and tumor progression. Therefore, optimizing the beneficial effects of the SASP on the TME while mitigating its harmful effects, combined with therapeutic strategies that incorporate anticancer drugs, senolytics, and senomorphics, offers a promising new approach for future clinical treatments.

## Author contributions

**Conceptualization:** Haijin Huang, Minhong Zhang.

**Formal analysis:** Siming Chen, Yitao Yang, Chunyun Fang.

**Funding acquisition:** Xiaojuan Zhong.

**Writing – original draft:** Kang Liu.

**Writing – review & editing:** Xiaojuan Zhong.
